# Imaging findings of retroperitoneal anastomosing hemangioma: a case report and literature review

**DOI:** 10.1186/s12894-022-01022-7

**Published:** 2022-05-22

**Authors:** Xing Xue, Mengchen Song, Wengbo Xiao, Feng Chen, Qiang Huang

**Affiliations:** 1 Department of Radiology, The First Affiliated Hospital, Zhejiang University School of Medicine, 79 Qingchun Road, Hangzhou, 310003 Zhejiang China; 2 Department of Radiology, Shulan (Hangzhou) Hospital, Zhejiang Shuren University School of Medicine, 848 Dongxin Road, Hangzhou, 310022 Zhejiang China

**Keywords:** Imaging, Retroperitoneal, Aanastomosing hemangioma, Case report

## Abstract

**Background:**

Anastomosing hemangioma is an uncommon benign vascular tumor that may be mistaken for a malignancy. The imaging findings of anastomosing hemangioma are not well provided from the previous reports. Herein, in the study, we discuss the imaging findings for one case of retroperitoneal anastomosing hemangioma.

**Case presentation:**

One 64-year-old female patient had a left retroperitoneal mass that was incidentally detected upon physical examination. A hypoechoic mass with abundant blood flow signals was found by US in the perirenal space. CT and MRI detected a large cystic and solid lesion in the left retroperitoneal space. Plain CT indicated that the internal density was uneven, and the pattern of enhancement was obvious and progressive. MRI-T2WI showed high intensity, DWI showed isointensity, and the mass also showed obvious progressive enhancement. Finally, anastomosing hemangioma was diagnosed via histopathological studies.

**Conclusion:**

As a rare and benign tumour, anastomosing hemangioma is easily misinterpreted. We suggest that the observation of “genitourinary tract related, well defined, hyperintensity or isointensity on T2WI, isointensity on DWI, and obvious progressive enhancement patterns likely to the vascular enhancement” may consider the diagnosis of AH.

## Background

Anastomosing hemangioma (AH) is an extremely rare and benign vascular tumor that was first described in the genitourinary tract by Montgomery and Epstein [1]. Cases of AH have been reported in most parenchymal organs and in a variety of soft tissue locations, but it mainly involves in the genitourinary tract [2]. Herein, we report on a case of retroperitoneal AH, which is rare and only 18 cases of AH occurring in the retroperitoneal space have been reported in the literature [1, 3–9]. Previous reports have mostly focused on the clinical and pathological features, and have provided scarce details regarding its imaging findings [3, 4]. Additionally, AH is easily misdiagnosed as other tumors, including malignant tumors, which has considerable effects on patients. As a result, knowledge of the imaging characteristics of this disease is very important. In this paper, we reported on the imaging characteristics that were observed for one case of retroperitoneal AH and retrospectively reviewed the imaging findings of previous studies.

## Case presentation

**Fig. 1:**
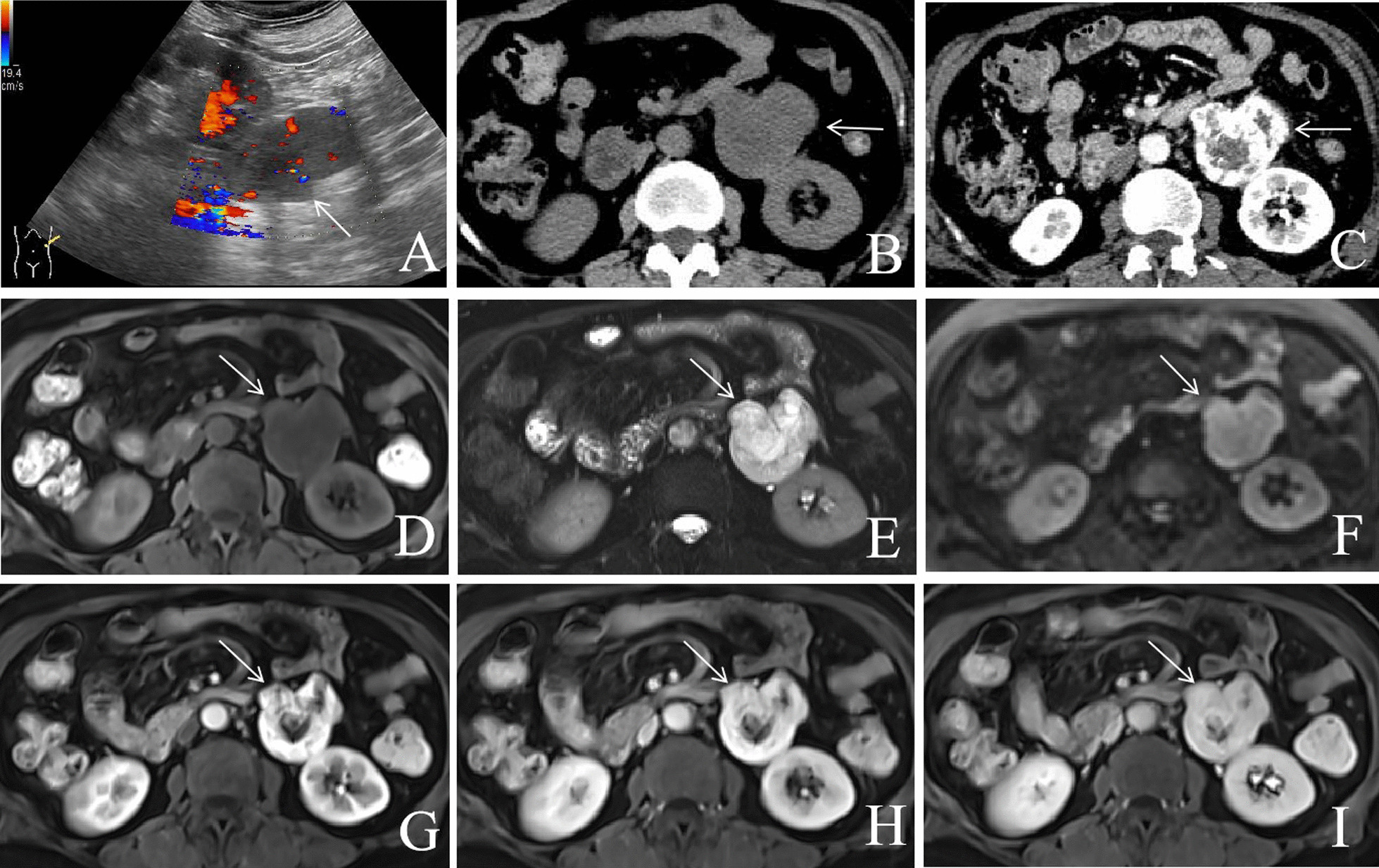
The lesion showed a hypoechoic mass with abundant blood flow signals in the perirenal space on US (**A**). Axial CT of the abdomen (plain scan and venous phase) showing a slightly low-density lesion (**B**) in the retroperitoneal space that was well-defined and with obvious enhancement (**C**). Axial pancreatic MRI showing a solid lesion with several cystic lesions, T1WI showed hypointensity (**D**), T2WI showed high-intensity (**E**), and DWI showed iso-intensity (**F**). The lesion had obvious progressive enhancement (**G**–**I**)

A 64-year-old Chinese female patient was observed to have a tumor lesion in the perirenal space by US, and was admitted to our hospital. The patient had hypertension, which was controlled well. Laboratory testing including routine blood examinations, serum electrolytes, and tumor markers were normal. The lesion exhibited a hypoechoic mass with abundant blood flow signals in colour Doppler flow imaging (CDFI) on ultrasonography (US) (Fig. 1). Contrast-enhanced computed tomography (CT) showed that the lesion was approximately 10.8 × 4.9 × 4.5 cm^3^ in size, well-defined and irregular, uneven and low density. Additionally, the pattern of enhancement was obvious and progressive. The plain CT value was approximately 17–36 Hounsfield units (HU). Contrast-enhanced CT scanning showed an inhomogeneous enhancement, and the CT value was approximately 111–173 HU in the arterial phase and 232–265 HU in the venous phase. The focal enhancement was likely to the vascular enhancement. Magnetic resonance imaging (MRI) also showed a well-defined and irregular, cystic and solid tumor lesion in the left retroperitoneal space. The T1-weighted image (T1WI) showed low intensity, the T2-weighted image (T2WI) showed iso- and high intensity, the diffusion-weighted image (DWI) showed iso-intensity. Likely to the pattern of enhancement of CT, the mass showed obvious and progressive enhancement on MRI. There was no evidence of invasion of the surrounding organs and no retroperitoneal lymphadenopathy been found. The preoperative diagnosis was diagnosed as being a retroperitoneal paraganglioma. Finally, endoscopic resection was performed after the surgical evaluation, and the histopathological diagnosis was anastomosing hemangioma. To date, the patient has been followed up for 24 months and there is no recurrence and transference of tumor.

## Findings of the operation and pathology

**Fig. 2:**
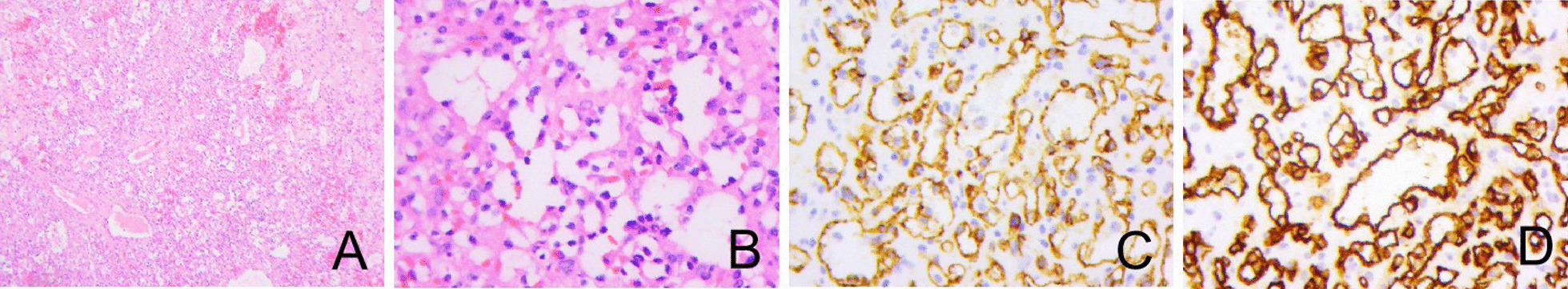
Microscopic examination with low (× 50) and high (× 400) power photograph. The low power photograph showed the tumor tissue was composed of tortuous and anastomotic capillary-sized vessels (**A**). The high power photograph showed wall of vessel was lined by spindle or oval endothelial cells, some of which protrude into the cavity. Additionally, the tumor cells were with evenly distributed euchromatin, typically bland appearing, and mitoses were scarce (**B**). Immunohistochemical studies showed the tumor cells expressed endothelial cells markers, which showed CD31 (**C**) and CD34 (**D**) were positive

During the operation, one tumor was found locate in the left retroperitoneal space, and it exhibited a clear boundary. The tumor had a complete capsule, and no lymph node metastasis. Regarding microscopy (Fig. 2), the low power photograph showed the tumor tissue was composed of tortuous and anastomotic capillary-sized vessels. The high power photograph showed wall of vessel was lined by spindle or oval endothelial cells, some of which protrude into the cavity. Additionally, the tumor cells were with evenly distributed euchromatin, typically bland appearing and mitoses were scarce. The immunohistochemistry results showed CD31 (+), CD34 (+), F8 (+), CgA (+), CKpan (+), FLi-1 (+), Syn (+), Ki-67 (+), S100 (+), and SMA (+).

## Discussion and conclusions

AH is an uncommon benign vascular tumor, especially occurred in retroperitoneal space. At present, only 18 cases of AH occurring in the retroperitoneal space have been reported in the literatures. Ours is the first radiological case report of AH including US, dynamic CT and MRI findings. From the previous literatures, most of the patients were incidentally identified without related symptoms, and no sex predilection has been previously reported [2]. The age of the affected patients ranges from 2 to 85-years-old (mean: 57.8 years). The youngest reported male patient had a 2.0 diameter tumor in the liver, whereas the oldest reported male patient had a 4.3 diameter tumor in the para-aorta [4, 10]. AH is mainly involved in the genitourinary tract (especially in the kidney), and it has been accepted as being an independent subtype of renal hemangioma in the 2016 new tumor renal classification [11]. From the previous literature, most AH cases were solitary. Bilateral and multiple lesions have also been documented [4, 8, 12–15]; however, they are rare. The sizes of AH are ranging from 0.1 to 14 cm, whereas over 65% of cases were no more than 3 cm in size, based on the limited information in the previous literature [3, 16]. Usually, AH is a solid lesion type, and cystic lesions can also be seen. The lesion is well defined but rarely encapsulated. In terms of the tumor capsule, most reports have shown no presence of capsules in AH. Similar to only a few previous reports [17–19], our case had a complete capsule. Unfortunately, the studies that have reported tumor capsules did not show imaging findings. Its shape can be roundish or irregular with lobulation [18, 20]. Furthermore, the internal density is uneven, and only a few cases have demonstrated the presence of fat or calcification [7, 21]. However, this is very rare. Consistent with the previous findings, our case involved elderly people who were asymptomatic, solitary, and cystic and solid type. But, this case had a complete capsule in pathological examination, which is unlikely to the most of cases.

On US, AH is usually observed as being a hypoechoic mass with a clear boundary, and some cases have shown rich blood flow signals detected by CDFI [5, 22, 23]. On CT, most of the lesions have hypointensity or isointensity in plain scanning, with a CT value of 27–35 HU [12, 23, 24]. Contrast-enhanced CT scanning showed that most lesions had obvious heterogeneous enhancement in the arterial phase and persistent hyperenhancement in the venous phase [10, 25–27]. Especially in cystic and solid lesions, there is strong nodular enhancement in the periphery [22, 28, 29]. Similarly, our case also exhibited peripheral nodular enhancement and centripetal filling enhancement. Not be mentioned in other literature, in this case, the peak value of enhancement was very high, which was likely to vascular enhancement. As the tumour tissue was composed of a vascular lumen and proliferated vascular endothelial cells. So, we thought that this help to explain the phenomenon. On MRI, internal intensity was uneven, T1WI showed low intensity, T2WI showed iso- to high intensity, and DWI showed isointensity to high intensity [6, 9, 10, 20, 28, 30, 31]. The MRI enhancement patterns were similar to those of CT, as there was also obvious heterogeneous enhancement in the arterial phase and persistent hyperenhancement in the portal venous and delayed phases [6, 28, 30]. Similarly to the MRI findings of some previous studies, our case was well-defined, solitary and exhibited a solid lesion with a small cystic lesion, as well as obvious progressive enhancement.

Considering the differential diagnosis, the paraganglioma or other malignant tumours were considered as possible diagnoses. In this case, several characteristics, including, irregular morphology, clear borders, heterogeneous density and signal, and obvious enhancement, were similar to those of a large extraadrenal paraganglioma. Large extraadrenal paragangliomas are often accompanied by necrosis and haemorrhage, sometimes with increased fluid levels caused by the haemorrhage. Additionally, the paraganglioma has a rich blood supply, and thickened tumour vessels can be seen around the mass or in the solid components [32, 33]. Another differentiated tumour is angiosarcoma, which is rare and tends to be aggressive [34].

AH is a benign tumour, and no recurrence has been reported. To date, the patient presented in this report has been followed for 24 months and has remained disease free. In a number of AH cases, after an accurate core biopsy diagnosis, the patients did not receive further treatment, and follow-ups showed no progression of the disease [4, 7]. From Zhou's study of 10 cases [2], none of the patients have evidence of recurrence, metastasis, and death, further indicating the innocent nature of AH. As a result, patient with identifying this tumour ahead may be suitable for follow-up and excessive surgical treatment may be avoided.

AH is an extremely rare and benign vascular tumour that can involve a large variety of internal sites. Correct diagnoses via imaging remain difficult to produce. We suggest that the observation of “genitourinary tract related, well defined, hyperintensity or isointensity on T2WI, isointensity on DWI, and obvious progressive enhancement patterns likely to the vascular enhancement” may need to be alert to the possibility of AH and may prevent unnecessary surgery for patients with this diagnosis.

## Data Availability

The clinical and imaging data are available from the corresponding author upon request.
